# Placing diverse knowledge systems at the core of transformative climate research

**DOI:** 10.1007/s13280-023-01857-w

**Published:** 2023-04-27

**Authors:** Ben Orlove, Pasang Sherpa, Neil Dawson, Ibidun Adelekan, Wilfredo Alangui, Rosario Carmona, Deborah Coen, Melissa K. Nelson, Victoria Reyes-García, Jennifer Rubis, Gideon Sanago, Andrew Wilson

**Affiliations:** 1 School of International and Public Affairs and Columbia Climate School, Columbia University, 420 West 118th Street, New York, NY 10027 USA; 2 Department of Sociology, Trichandra Multiple Campus, Tribhuvan University, Kirtipur, Kathmandu, 44605 Nepal; 3 Global Environmental Justice Research Group, School of International Development, University of East Anglia, Norwich, UK; 4 Department of Geography, Faculty of the Social Sciences, University of Ibadan, Ibadan, Nigeria; 5 Department of Mathematics and Computer Science, College of Science, University of the Philippines Baguio, Governor Pack Road, Baguio, 2600 Philippines; 6 Department of Anthropology of the Americas, University of Bonn, Bonn, Germany; 7 Center for Integrated Disaster Risk Management (CIGIDEN), Pedro Torres 460, apt. 405 B, Santiago, Chile; 8 Department of History and Program in the History of Science & Medicine, Yale University, 320 York St, New Haven, CT 06511 USA; 9 School of Sustainability, College of Global Futures, Arizona State University, 777 E. University Dr, Tempe, AZ 85281 USA; 10 ICREA and Institute of Environmental Science and Technology, Universitat Autònoma de Barcelona, Cerdanyola del Vallès, 08193 Barcelona, Spain; 11 Green Climate Fund, Incheon, 22004 Republic of Korea; 12 Pastoralists Indigenous NGO’s Forum (PINGO’s Forum), P.O.Box 14437, Sakina kwa Iddi, Arusha, Tanzania; 13 School of International and Public Affairs, Columbia University, 420 West 118th Street, New York, NY 10027 USA; 14 Global Policy Lab, UC Berkeley, Berkeley, CA USA

**Keywords:** Climate, Co-production, Decolonization, Indigenous, Knowledge, Transformation

## Abstract

We argue that solutions-based research must avoid treating climate change as a merely technical problem, recognizing instead that it is symptomatic of the history of European and North American colonialism. It must therefore be addressed by decolonizing the research process and transforming relations between scientific expertise and the knowledge systems of Indigenous Peoples and of local communities. Partnership across diverse knowledge systems can be a path to transformative change only if those systems are respected in their entirety, as indivisible cultural wholes of knowledge, practices, values, and worldviews. This argument grounds our specific recommendations for governance at the local, national, and international scales. As concrete mechanisms to guide collaboration across knowledge systems, we propose a set of instruments based on the principles of consent, intellectual and cultural autonomy, and justice. We recommend these instruments as tools to ensure that collaborations across knowledge systems embody just partnerships in support of a decolonial transformation of relations between human communities and between humanity and the more-than-human world.

## Introduction

To focus on solutions, as the term “solutions-oriented research” suggests, is to risk prioritizing ends over means; we argue that the research *process* is as important as the output. For reasons that are both epistemic and ethical, we hold that research on climate change must facilitate genuine partnership across knowledge systems. We argue as well that such partnerships can be built only as part of larger, more encompassing political shifts. Indigenous Peoples have long pressed for recognition and respect of their knowledge systems as part of their struggles for self-determination; local communities around the world have engaged in parallel efforts, as well. International environmental organizations are increasingly attempting to engage with non-Western knowledge systems, but too often they do so in ways that detach information gathered from this engagement from the worldviews, values, embodied practices, and relationships that are both definitive of and vital to those knowledge systems. Only by fully recognizing this holistic quality of knowledge systems can climate research begin to point beyond short-term fixes, toward a transformation of attitudes, of ways of life, and, ultimately, of power structures. Only by confronting the deep histories of the unjust—and often exploitative and violent—relations between the societies and peoples that hold these knowledge systems can climate research undo the forces that block this transformation.

Our recommendations for transformative change presume that institutions of knowledge-making and institutions of governance do not change independently of each other. In other words, epistemic justice–full, fair inclusion and participation of different knowledge systems–is inseparable from social and political justice. As Sheila Jasanoff has theorized, science and society “co-produce” each other, meaning that the practice of science inevitably reflects the power relations of the existing social order, and when people set the rules of knowledge-making, they are also defining social norms (Jasanoff 2004). This usage contrasts with the way that “co-production” is commonly used in climate science today to designate collaboration between scientists and other groups of experts and knowledge holders, often limited to short-term projects. Transformative change to solve the climate crisis will depend on careful attention to these interactions between knowledge and power.

Our argument takes three steps. First, we review definitions of knowledge systems and recent empirical research on knowledge systems. We call for a fuller view of knowledge systems themselves. We note the persistent social and political inequalities that marginalize certain knowledge systems. Second, we review the positions on knowledge systems that are taken by the social and political institutions that frame climate research and action. We propose that scientific research remains inflected by the history of European colonialism; research that aims at transformative change must begin by decolonizing the research process itself. We emphasize how undoing the epistemic injustice associated with colonialism means rethinking the divide between knowledge and values that currently structure international scientific and policy institutions like the UNFCCC and IPCC, as we argue in "Transformative change" and "Recommendations" sections. Finally, we call for concrete, accountable steps to achieve genuine partnerships. We show in the final sections of this Perspective that calls to pluralize the research process are meaningless without concrete governance mechanisms to guarantee that collaboration proceeds with due respect for the cultures, worldviews, goals, and rights of Indigenous Peoples and of local communities. Our Perspective culminates in a list of instruments whose use we recommend in order to ensure just partnership across knowledge systems for transformative change. Not all these instruments are novel—some were first proposed decades ago—but we note that they have been put into practice at best in an incomplete and piecemeal fashion; our contribution therefore is to emphasize the necessity of full implementation of the full set of these instruments. No set of instruments can on their own guarantee transformative change, since instruments can often be abused by the powerful, but the absence of instruments makes it unlikely if not impossible.

## Knowledge systems

In our discussion, we rely on the general term “knowledge systems,” since it signals that the scope of knowledge is broader than facts, hypotheses, and observational techniques, and since it is commonly used in research publications, reports, and assessments. The Intergovernmental Panel on Climate Change (IPCC) recently defined knowledge systems as “sets of interacting agents, practices and institutions that organize the production, transfer and use of knowledge” (Petzold et al. 2020). The IPCC goes on to state that “this definition emphasizes the social nature of knowledge and the importance of the link between knowledge and action, rather than presenting knowledge simply as information about past, present and future states of the world.” A similar definition of this term can be found in a glossary of the Intergovernmental Science-Policy Platform on Biodiversity and Ecosystem Services (IPBES 2017).

However, we recognize that many researchers, including Indigenous scholars, prefer the term “ways of knowing” (Basso 1996; Aikenhead and Michell 2011; Cochran et al. 2013; ICC 2016; Ferguson and Weaselboy 2020; Nelson 2020; Chiblow and Meighan 2022). Like “knowledge systems,” the term “ways of knowing” conveys that knowledge holders adapt to changing conditions and realities. The term “ways of knowing” has the advantage of signaling that knowledge is not a list of doctrines but rather a coherent, dynamic set of practices, values, and worldviews. The term indicates (perhaps more directly than “knowledge systems”) that knowledge can simultaneously be rooted in and cohere to culturally specific ethical principles such as respect for and reciprocity with nonhuman life.

In particular, Indigenous Peoples have developed a wide variety of distinctive ways of knowing, often termed Indigenous knowledge systems or Indigenous People’s knowledge systems, that are embedded in and proceed from local ecosystems and ecological processes that are encoded in distinct Indigenous languages (Salmon 2000). Potawatomi scholar Robin Wall Kimmerer often calls this the “grammar of animacy,” as Indigenous languages often emerge from the sounds of the landscape (Kimmerer 2013). This phrase indicates that many Indigenous languages, and thus Indigenous Peoples’ epistemologies and ontologies, understand the world as a series of processes, flows, and actions more than static objects or relations (Ferguson and Weaselboy 2020; Nelson 2020). Additionally, Indigenous oral stories and complex narratives often outline spiritual values, ethical and pragmatic teachings, and ways of being that literally and metaphorically link worldviews to place-based environmental knowledge (Aikenhead and Michell 2011). These ways of knowing are not neutral or objective but are rooted in a value-rich participatory relationality. As Keoki Kanakaokai, an Indigenous natural resource manager in Hawaii, explains, “We take into consideration more than just Western empirical science but also incorporate Traditional Ecological Knowledge [and] traditional ways of knowing. In particular, we value things like *kilo*, the Hawaiian concept of observation and building relationships with our resources” (Cartier 2022); for other discussion of Traditional Ecological Knowledge, see Nelson and Shilling, eds. (2018) and Wildcat (2023). Similar points can be found in some earlier work of academic anthropologists, which examines complex systems of understandings and engagements with the natural world, drawing on a variety of approaches, including phenomenology (Ingold 2011), practice theory (Ellen 2004), structuralism (Lévi-Strauss 1966), and political ecology (Sillitoe 2007); in different ways, this earlier work, though often situated within disciplinary frameworks that posit Indigenous knowledge systems as objects of scientific research, opens some discussion of Indigenous knowledge systems and local knowledge systems as full, complex, and autonomous alternatives to scientific knowledge. At the same time, it is important to acknowledge that, through the long history of colonialist epistemic extractivism, Indigenous knowledge systems have informed the contents of both “Western” sciences and theories of scientific knowledge (Haraway 2016; Graeber and Wengrow 2021).

As suggested above, Indigenous knowledge has been defined in a number of related ways, as definitions depend on the right to self-determination (van Bavel et al. 2022). We follow the Indigenous scholars cited above in giving descriptions of Indigenous ways of knowing rather than providing a simple glossary-style definition, since such definitions often fall short, because Indigenous languages often cannot be readily translated into English and, moreover, because such definitions can be used against Indigenous self-determination.

We note that the discussions of local knowledge are less extensive. The IPCC defines local knowledge as “the understandings and skills developed by individuals and populations, specific to the places where they live. Local knowledge informs decision-making about fundamental aspects of life, from day-to-day activities to longer-term actions. This knowledge is a key element of the social and cultural systems that influence observations of and responses to climate change; it also informs governance decisions” (IPCC 2018). In addition, we recognize that some sources distinguish between place-based and activity-based local knowledge systems; the latter, sometimes called practitioner knowledge systems, have been defined as “the pragmatic, practice-based knowledge that comes from the regular exercise of craft or professional work” (New et al. 2022).

Both terms—knowledge systems and ways of knowing—can help us see what science and Indigenous knowledge have in common (Watson-Verran and Turnbull 1995). As an inescapably human enterprise that adjudicates among different knowledge claims, science inevitably embeds values. These include both “cognitive” values such as simplicity and consistency as well as “non-cognitive” or social values (Oreskes 2020). Feminist philosophers of science have shown that even this distinction is misleading. For instance, the choice to value the “simplicity” or “elegance” of an explanatory theory—say, the theory that migration is caused by climate change—is often a choice to overlook the complexity of underlying conditions, thus eliding social, economic, and political circumstances that shape vulnerability. Indeed, the goal of “value-free” science—an ideal implicit in the IPCC’s founding ambition to produce “policy-relevant but policy-neutral” reports—is misguided. In the early days of climate change research, most scientists maintained a posture of political neutrality out of fear of losing professional credibility, and this posture contributed to political inaction (Howe 2014). Solutions-oriented research must recognize that social values play legitimate, vital roles in science: inevitably, they shape research agendas and inform judgments about whether a conclusion is adequately supported by evidence (Longino 1996; Douglas 2000). We call for partnerships to make explicit the different values that guide them (Longino 1990; Richardson 2015).

Though much recent work on knowledge systems and on ways of knowing supports the full inclusion of diverse knowledge systems in solutions-oriented climate research and other settings (see "Transformative change" section), the recognition given to Indigenous knowledge systems and local knowledge systems often remains limited. Too often, scientists have misrepresented Indigenous knowledge systems and local knowledge systems as simple repositories of empirical observations (Nakashima et al. 2012; Granberg and Glover 2014). A review of 81 studies of research that sought to engage substantially and deliberately with multiple knowledge systems in climate adaptation found that the dominant pattern was for scientific researchers to treat Indigenous knowledge and local knowledge as sources of data or information that could be used, in isolation from the contexts in which they were produced, to fill gaps in scientific frameworks and models (Klenk et al. 2017).

Two contrasting studies of research on crop diversification may be taken as examples of different types of work that draws on diverse knowledge systems. This agricultural practice, widely used by Indigenous Peoples and local communities, has often been recognized for its potential to reduce the food security risks that have expanded conditions of climate change (Bezner Kerr et al. 2019). A study of Indigenous farmers in the Indian Himalaya (Shukla et al. 2016) may be taken as an example of a less inclusive approach to Indigenous knowledge systems. The authors count the frequency of the practice by households and correlate its use with the villagers’ stated perceptions of changes in precipitation and temperature. They deemed the villagers’ perceptions, based on many generations of observations, to be correct when they coincided with data from the nearest meteorological station (some 40 km away, at a lower elevation, and with data available only for the last 30 years). They reviewed the potential compatibility of crop diversity with modern agronomic practices and financial instruments, and evaluated its potential transferability to other settings. In contrast, other studies offer a fuller view of crop diversification within broader ways of knowing. For example, Rarai et al. (2022), discussing Vanuatu in the western Pacific Ocean, presents crop diversification as a complex set of activities centered in a communitarian, relational worldview that stresses the mutual responsibility of humans, landscapes, and spirits. The author team, led by an Indigenous researcher, explores more broadly the potential for this practice to operate within partnerships that link Indigenous knowledge holders with modern adaptation projects at the national level and with scientific knowledge systems that provide projections of future climate conditions. Though both studies consider crop diversification to be positive and argue that it should be supported, Shukla et al. (2016) suggest that it could be transferred as an isolated practice that would form one component of expert-driven projects, while Rarai et al. (2022), who note that such projects often do not promote long-term change, indicate instead that diversification could more effectively be fostered by promoting local languages, maintaining cultural practices, and supporting village-based economies rather than commercial agriculture.

This contrast suggests the range of approaches to the interaction of knowledge systems. Petzold et al. (2020) note that many studies, like Shukla et al. (2016), focus only on specific practices that align with specific economic agendas, and seek to include these practices alone into responses to climate change. They argue that this kind of recognition continues to perpetuate a top-down approach that subordinates Indigenous knowledge systems and local knowledge systems to scientific knowledge systems. This approach also decontextualizes these knowledge systems because, by focusing on observations and practices, it omits components of the system, such as governance, values and laws, and worldviews, that are relevant to understanding and responding to the climate crisis (Carmona et al. 2022a). More broadly, this pair of studies can serve to raise a question: what are the conditions of the research process that can distinguish in advance how successful a study will be in achieving the goals of sustainability, inclusion, and equity?

Perspectives similar to Rarai et al. (2022) rest not only on epistemic justice (the full and equitable inclusion of diverse knowledge systems) in isolation but also on the procedural and distributive justice issues that assure equitable governance (Kidd et al. 2017). For example, Latulippe and Klenk (2020) reject extractive approaches that treat Indigenous knowledge as “data that can be aggregated and understood in abstract and universal form.” They go on to show, through an examination of cases of Indigenous stewardship of water and fish in Ontario, Canada, that Indigenous ways of knowing cannot be detached from specific landscapes; from cultural and spiritual practices that form communities of humans, animals, and spirits marked by relationships of recognition and care; and, ultimately, from Indigenous self-determination. The authors suggest that specific knowledge techniques, like forms of observation of water quality or fish populations, cannot be detached from their contexts and incorporated into packages of solutions that can then be transferred to other settings—a point that underscores the importance of assuring Indigenous land rights and self-determination as preconditions for epistemic justice. In a similar vein, Nkuba et al. (2020), examining a variety of agricultural practices in western Uganda, show that farmers pay attention both to Indigenous weather knowledge that circulates within local communities and scientific forecasts that they receive in local languages by radio; their responses reflect not only the credence that they place on these knowledges and the identities associated with them, but also with the social systems that govern—and often limit—access to land, labor, and agricultural inputs. The authors suggest that expanding the scope of farmers’ responses to climate change thus rests not only on supporting the access to diverse forms of information, but expanding the capacity of individuals, households, and communities to govern their lands.

For collaboration and mutual exchange between diverse knowledge systems to contribute to climate solutions, the scientific community will need to transcend and overcome widespread biases against Indigenous knowledge and local knowledge. This task is often slow and filled with challenges, as shown in a recent review of the team that prepared the Northwest regional chapter for the Fourth U.S. National Climate Assessment (Roesch-McNally et al. 2020). The authors had a mandate to work with “diverse participants'' and to acknowledge “multiple ways of knowing, including local and Indigenous knowledge.” They note that this assessment represented an advance over prior assessments in this regard, and reported that this diversity of participants led to “the shift in the Northwest chapter’s framing from the authors’ original framing, articulated before the stakeholder engagement meetings, focused around subregional cultures and impacts (i.e., coastal, western lowlands, mountains, and inland) to a collective regional identity and values-based framing (e.g., outdoor recreation, rural food systems, hunting and fishing, and tribal and Indigenous cultures)” (340). This diversity also permitted fuller discussion of climate impacts, including ones that were less fully reported in the scientific literature. However, Roesch-McNally et al. (2020) stated the need for deeper, more sustained engagement with diverse authors and stakeholders and fuller incorporation of epistemic plurality and different knowledge systems. They noted barriers that limited the effectiveness of the stakeholder meetings, which were planned and organized by agency staff rather than in collaboration with others, and that they were held in cities with large airports, rather than in smaller sites where stakeholder groups and communities reside. They noted as well the challenges of building effective processes within the time frames mandated by the national government. They observed that the regulations that structure national climate assessments prioritize evidence from scientific knowledge, especially articles in peer-reviewed journals, over evidence from other ways of knowing. Though the authors do not use the term “decolonization,” their discussion parallels a claim of this article: decolonization is a long process that must proceed by building trust and by recognizing a rights-based approach that spans political and legal rights, economic, social and cultural rights, and human rights more broadly.

Despite these challenges, the potential for collaboration of diverse knowledge systems is demonstrated in a growing body of literature, much of it quite recent. In Tanzania, Indigenous People are currently working with the Tanzania Meteorological Authority on weather forecasting to inform climate decision-making processes at local and national scales (Climate Action Network Tanzania 2022; Lusiru and Malekela 2022). Likewise, the work of Kassam, Haag, and colleagues in mountainous villages in Tajikistan integrated scientific data with community observations in local climate trends to understand the scope and impact of climate change in the livelihoods and food systems in these upland areas. Their study showed that an integrative assessment using both scientific and local knowledge systems allowed for an enriched understanding of local climate trends by reducing existing uncertainties, providing new information, and introducing unforeseen perspectives (Haag et al. 2021; Kassam et al. 2021). In Australia and California, Indigenous fire management practices are now widely acknowledged and are incorporated into a multi-faceted solution to wildfire issues (Erickson and Hankins 2014; McKemey et al. 2021). Likewise, Fiji recently recognized customary land tenure and supported traditional meetings—attended by men and women of all ages–as a form of governance (Eräsaari 2015; Harris et al. 2020; Tomlinson 2020; Gard 2021). In this setting, village members, led by male elders, planned the relocation of coastal villages impacted by coastal erosion and saltwater intrusion, drawing both on Indigenous knowledge and on scientific information contributed by national meteorological services and other science-based agencies (McMichael et al. 2019). The decisions not only supported livelihoods and use of terrestrial and marine resources, but maintained cultural values and connections to ritual sites, providing an example of solutions based research that illustrates the partnership of diverse knowledge systems (Eräsaari 2015; Harris et al. 2020; Gard 2021). In Canada, the federal government has established three high-level bilateral roundtables with First Nations, Métis, and Inuit to discuss the design and implementation of climate policy (Reed et al. 2021). Among other things, this collaboration resulted in an annex on Indigenous Climate Action with three sections in the Canadian Nationally Determined Contribution to the United Nations Framework Convention on Climate Change (UNFCCC), drafted by representatives of the three groups (Canada’s 2021* Nationally Determined Contribution Under the Paris Agreement*, 2021). A study of cases of Indigenous decision-making in Peru, Pakistan, China, and the U.S. shows that Indigenous-led planning to adapt to climate-change impacts can receive support from science-led government agencies and NGOs when community rights to manage resources are acknowledged (Orlove et al. 2020).

Our discussion now turns from an overview of diverse knowledge systems and cases of collaboration to a review of the ways that international climate agreements and organizations frame diverse knowledge systems. We note significant progress in recent years toward fuller recognition of the value of these knowledge systems—and we also note the persistence of barriers that impede full, just partnerships. In the section after next, we call for transformations to make pluralism, inclusion, and epistemic justice more than general goals—or, perhaps, appealing slogans—and instead ensure that they become realities.

## The position of diverse knowledge systems within international climate agreements and institutions

The way in which international climate and environment assessment processes approach diverse knowledge systems and the forms of climate research that are prioritized influence the solutions pursued for mitigation and adaptation. We therefore explore how Indigenous knowledge systems and local knowledge systems have been considered within key reports from the past 20 years from the IPCC and the UNFCCC, as well as UNESCO, IPBES, and the Convention on Biological Diversity (CBD), which also consider climate and climate-adjacent issues; we review the instruments put in place to facilitate the inclusion of diverse knowledge systems and interactions between them. We perform a narrative synthesis of the major reports from these orgranizations to critically analyze how diverse knowledge systems are conceptualized, what principles are articulated for their recognition, and the form and extent of collaboration involved and advocated.

Overall, the importance of drawing on diverse knowledge systems to address climate change, along with other complex sustainability problems, took a major step forward with the Rio Declaration of the 1992 Earth Summit, an event marked by significant Indigenous mobilization (Nakashima et al. 2017). The Declaration, which set up the UNFCCC and the CBD—as well as the UN Convention to Combat Desertification—lays out the principles by which the Conventions would be formed; Principle 22 of the Rio Declaration states that Indigenous people and their communities and other local communities have a vital role in environmental management and development because of their knowledge and traditional practices. It further indicates that States should recognize and duly support their identity, culture and interests and enable their effective participation in the achievement of sustainable development. Building on this declaration, UNFCCC and the CBD—as well as the IPCC, UNESCO, and IPBES—have attended significantly to these knowledge systems. Some of their documents emphasized characteristics that are shared across many Indigenous knowledge systems and local knowledge systems, such as worldviews that rest on deep links between nature and culture, appear widely acknowledged (Tengö et al. 2017; Petzold et al. 2020; UNESCO 2020). Consideration of Indigenous knowledge systems and local knowledge systems in these reports and other documents goes beyond discussions of their vulnerability to emphasize their crucial governance and management role and the need for effective partnership with scientific knowledge systems to address climate change and biodiversity loss. Since the early 2000s, a number of specific principles regarding the treatment of different knowledge systems have become widely and consistently articulated across international climate and biodiversity governance processes. These include Free, Prior, and Informed Consent (FPIC); the recognition of diverse worldviews, knowledge systems, and customary institutions; and respect for rights (Ituarte-Lima et al. 2018). Despite this, numerous issues, such as data sovereignty, remain unaddressed (Reyes-García et al. 2022).

This attention to diverse knowledge systems has carried across various platforms (Nakashima et al. 2017). One example is provided by UNESCO’s Local and Indigenous Knowledge Systems (LINKS) program, established in 2002, which connects Indigenous Peoples and local community knowledge holders to IPCC Working Groups. Another is provided by the Nairobi Work Programme of the UNFCCC, which included from the outset an emphasis on Indigenous and local knowledge, has reviewed available tools and best practices for their incorporation into adaptation (Nakashima and Nilsson 2006; Thaman et al. 2013). The Green Climate Fund, which was established by the UNFCCC in 2010 as an operating entity of the Financial Mechanism of the Convention, adopted its Indigenous Peoples Policy in 2018 and operationalized its Indigenous Peoples Advisory Group in 2022. More recently, the 2021 Glasgow Pact emerging from the 26th Conference of the Parties to the UNFCCC (COP26) “urges Parties to actively involve indigenous peoples and local communities in designing and implementing climate action,” also noting their “important role” in “effective action on climate change.” Although the progress made at UNFCCC COP27 in 2022 was relatively minor—the references to Indigenous issues in the cover decision were minimal and the Indigenous demand of including Indigenous rights in Article 6, related to emission reduction targets, was rejected—we highlight one point of progress: the assignment of one position for Indigenous Peoples organizations on the advisory board to the newly created Loss and Damage Fund (UNFCCC 2022a). For comparison, further advancements were seen at the CBD COP15 in 2022 in Canada: the new Kunming-Montreal Global Biodiversity Framework makes strong references to Indigenous Peoples’ rights, recognizes their territories, and promotes consideration of their knowledge and their participation during implementation.

However, despite long-standing progressive principles about diverse knowledge systems and their consistent inclusion within environmental policy objectives and social safeguard standards for programs, progress has been slow, whether in site-based research informing climate policy, the local implementation of projects, the inception and design of programs at regional or wider scales, national policy processes and assessments like Nationally Determined Contributions toward climate change mitigation and adaptation, or the structures and functioning of international climate and environmental negotiations (Nakashima et al. 2017; Kuyper et al. 2018; Nelson and Madsen 2018; Klinsky 2019; Mustonen et al. 2021; Carmona et al. 2022b; McAllister et al. 2022). In general, across scales, scientific knowledge systems have continued to dominate, while the aspiration that those processes should involve collaborations based on meaningful intercultural recognition in appropriate forums remains distant (Ulloa 2017; Okereke 2018).

Regarding climate research, methodologies supporting the co-production of knowledge have advanced and have been increasingly applied (Gram-Hanssen et al. 2021). Within IPCC assessment processes, the Fifth Assessment Review (AR5) (2013/14) explicitly described the need to consider diverse knowledge systems in climate research (Petzold et al. 2020). Since then, specific examples of Indigenous knowledge systems from across the world and their contribution to climate change adaptation or mitigation have increasingly featured in IPCC reports (van Bavel 2021). However, the prevailing narrative continues to emphasize the vulnerability of Indigenous Peoples and local communities to climate change, with the IPCC’s AR6 Working Group II report of 2022 being the first to specifically highlight the historical processes of colonization, marginalization, and disruption of knowledge systems that underlie that vulnerability (Belfer et al. 2017; Mustonen et al. 2021). However, despite greater attention to Indigenous knowledge systems and Indigenous scholarship, the IPCC assessment methods do not include any requirement to respect Indigenous data governance protocols (Krug et al. 2020), which could ensure Indigenous Peoples’ rights to protect knowledge are fulfilled, as stipulated by the United Nations Declaration on the Rights of Indigenous Peoples (UNGA 2007).

Regarding climate change governance processes, the participation of Indigenous Peoples and local communities is often assessed to be inadequate or symbolic rather than full and effective (Brugnach et al. 2017). For example, the Local Communities and Indigenous Peoples Platform (LCIPP) of the UNFCCC has been operational only since 2018. LCIPP provides recognition of Indigenous Peoples’ knowledge, values, and practices as part of climate solutions. For the first time in 2021—at COP26 in Glasgow—the Indigenous Peoples’ Pavilion was located in the same area as delegates and formal processes, rather than in a separate exhibition zone. Fundamentally, discussions around collaboration among diverse knowledge systems tend to focus on the equitable distribution of financial costs and benefits alongside participation, rather than opening up to epistemic and historically grounded forms of justice, including anthropogenic drivers of climate and ecological crises (Klinsky 2019). The mainstream mechanisms and responses proposed, supported, and implemented through those processes most often fall far short of the biocultural approaches called for by Indigenous Peoples, local communities, and their representatives that would see local values, territories, and customary institutions embedded within climate action and viewed as a key vehicle for achieving climate and environmental outcomes and ensuring resource rights, intellectual property rights, and data sovereignty of Indigenous Peoples and local communities (Maffi 2018).

One factor enabling the gap to persist between the principles for and actual experiences of implementation is the limited efforts to design and put in place specific policy instruments through which those principles and standards can be met and accountability upheld. For example, although FPIC is consistently included as a principle or objective, implementation processes often fall short of meeting requisite standards and are minimally accountable to the extent that FPIC is seldom treated as pivotal to whether or how a project proceeds. For example, the Philippine government established FPIC as national law in 1997 Indigenous Peoples’ Rights Act (IPRA) in the Philippines; this act requires all “development projects and programs” whether by government or private entities, to go through FPIC. Nonetheless, Daytec-Yañgot documented many cases of violations of foreign-owned mining companies in the Philippines with the help of the Department of Environment and Natural Resources from 1999 to 2005. These firms ignored the FPIC enshrined in national law or even manipulated it to justify operations despite documented opposition of affected communities (Daytec-Yañgot 2012). Similarly, Talamayan reported similar violations of commitments to FPIC in recent and ongoing cases of proposed dam construction in the Philippines, including the Kaliwa dam that will affect the Dumagat-Remontado Indigenous Peoples, in southern Luzon, where construction is set to begin despite local opposition (Talamayan 2020).

Indeed, there are multiple definitions of FPIC, from the relatively comprehensive description within the non-binding United Nations Declaration of the Rights of Indigenous Peoples (which includes criteria such as redress for cultural, intellectual, religious, and spiritual property taken without FPIC) to less stringent definitions associated with project funders such as the Green Climate Fund’s Indigenous Peoples Policy (UNFCCC 2022b) or the World Bank’s Environmental and Social Standards (which focus on obtaining “broad community support” for a current specified intervention) (Raftopoulos and Short 2019). In a similar fashion, principles aspiring to recognize knowledge systems, customary institutions, and traditional practices are commonly listed in policies, safeguards, and objectives, yet clear tools and instruments are not always provided or used for identifying, documenting, and respecting rights associated with relevant institutions as standard practice. This lack of consistent definitions reflects the political obstacles faced by those who call for full justice and recognition. Many national government policies and practices fall short of recognizing Indigenous knowledge systems and local knowledge systems and also fail to assure full and effective participation. These gaps create the need for further work by civil society organizations and international bodies that join with Indigenous Peoples and with local communities to put effective instruments in place (Walter and Urkidi 2017). In sum, we note the persistence of limits to epistemic justice, including the persistence of institutional and governance structures that impose such limits. These conditions lead us to call for transformative change, discussed in the next section.

## Transformative change

As argued above, we observe, as many have, that action to date on climate change has been insufficient to address the existential threat created by current and projected impacts (Huggel et al. 2022). We therefore call for a transformation of climate research as a necessary component of solutions to the climate crisis. We join with others in discussions about climate change who use the term “transformative change” and the associated opposition between transformative and incremental change to signal the necessity of large-scale fundamental shifts throughout society rather than smaller adjustments (O'Brien et al. 2021; Orlove 2022); however, we are aware of the concerns that have been raised about the term—its association with neo-liberal policies or with top-down, managerial approaches (O’Brien 2012), its potential to silence and marginalize voices (Blythe et al. 2018), and its inherent ambiguity, which allows it to be put to many different purposes (Kasdan et al. 2021). We recognize as well that some researchers argue that incremental actions can serve as steps toward transformation, while others suggest that they undermine transformation by diverting effort away from fundamental change or by reinforcing existing path dependencies (Jagannathan et al. 2020).

We emphasize that overall transformation entails several specific transformations. We note our discussion draws inspiration from arguments that have been made by several Indigenous scholars. Their work underscores the deep and ongoing history of colonization that creates environmental harms and that expropriates and marginalizes Indigenous knowledge systems (Todd 2016; Davis and Todd 2017; Whyte 2017). Tāłtān scholar Candis Callison argues that increasingly destructive fires in Canada, the U.S., and Australia are not only the result of climate change, but also of the interaction of the climate crisis with flawed land management resulting from the often violent exclusions that accompanied colonialism (Callison 2021). In that sense, addressing wildfire, an impact and driver of the climate crisis, requires reshaping the relations of people and nature, such as the restoration of land and resource rights to the Indigenous Peoples and to the local communities from whom they were stripped. Indigenous understandings of humans as an integral component of nature connect ecological knowledge to social, cultural, and spiritual values (Lyver et al. 2017; Liboiron et al. 2018; Coscieme et al. 2020) that can guide transformative change.

Firstly, we call for solutions-based research to more broadly define the problems to be solved. Such efforts can advance by fully incorporating the deep-rooted knowledge possessed by Indigenous Peoples and local communities, arising from their close observations of and engagement with the natural world (Orlove et al. 2000; Fernández-Llamazares et al. 2015; Reyes-García et al. 2021; Schlingmann et al. 2021). Moreover, because Indigenous knowledge systems and local knowledge systems are fundamentally shaped by different worldviews, cosmologies, and ontologies that posit distinct ways of relating with humanity and nature, these knowledge systems can play a fundamental role in providing alternatives for new forms of governance and more-than-human values (O’Brien and Sygna 2013; Lam et al. 2020; Nightingale et al. 2020; Chakraborty and Sherpa 2021; Vogel and O’Brien 2021). These alternatives can lead to just partnerships based on mutual recognition and respect between the peoples, communities, and societies of knowledge holders (Adger et al. 2013; IPCC 2014; Jones et al. 2014; Yeh 2016; Klenk et al. 2017; Petzold et al. 2020).

While scientific knowledge systems have contributed to the mitigation of and adaptation to climate change, their narrow, technical, and dominating approach to problem-solving often marginalizes solutions held by other knowledge systems, limiting the participation and contributions of these systems at a time when they are urgently needed. By contrast, the gravity of the climate crisis has prompted calls for research that supports transformative change—a fundamental reshaping of human relationships to the non-human world (O’Brien and Sygna 2013; Vogel and O’Brien 2021). The IPBES Global Assessment (Díaz et al. 2015), the latest IPCC report (IPCC 2022), and the joint IPBES–IPCC report (Pörtner, et al. 2021; Pascual et al. 2022) acknowledge that transformative—rather than incremental—change is needed to address the climate and biodiversity loss crises in socially just ways.

Secondly, as mentioned earlier, our recommendations for transformative change are based on the recognition that institutions of knowledge-making and institutions of governance do not change independently of each other. Transformative change to solve the climate crisis must rest on close attention to these interactions between knowledge and power.

Thirdly, our view of transformative change includes not only a look ahead to a future different from our present reality, but also a sustained look back to the long history of exploitation of peoples and environments by European empires and their successor states. This sobering view leads us to call for decolonization, involving the recognition of past violence and exclusion as well as acts of restitution. Climate change underscores the importance of the broad challenge of decolonization, bringing it into wider view internationally, nationally, and locally as its unjust consequences grow more evident. On the one hand, this immense task of decolonization means that recommendations for more diverse knowledge systems cannot be made in a facile manner, as if these changes were easy to achieve; on the other hand, the immense task of confronting the climate crisis creates support for wider coalitions and more inclusive efforts.

These three points correspond only partially with certain other views on transformative change. Both the IPCC and IPBES speak of transformation in terms of systemic transitions and include shifts in governance and values in order to address the underlying drivers of climate and environmental crises (IPCC 2022). We present a vision of an inclusive process for the making and implementation of new knowledge—one that foregrounds the roles of values and the links between knowledge and governance. However, unlike the definitions of transformation offered by the IPCC and IPBES—which focus on large-scale systems—we emphasize that change must occur at all scales. Transformation requires, in particular, new ways of linking international research to local concerns, as well as new ways of bringing local and Indigenous knowledge to bear on global governance, including the development and implementation of adaptation and mitigation measures (Renn 2020; PICC 2022)***.***

## Recommendations

We suggest a path toward the full inclusion of diverse knowledge systems in climate research. This recommended path begins with a statement of the general goals to support such inclusion, continues with three specific recommendations to achieve these goals, and closes with a review of specific instruments that have been developed to assure inclusion of knowledge holders (primarily Indigenous Peoples but also with applicability to local communities) across a number of domains. We conclude by noting that progress to date, though significant, remains incomplete, so that further work remains to ensure that instruments such as these are put into full practice. We acknowledge that there have been many similar calls for transformation, but we emphasize that the time for action is now in light of the urgency of the climate crisis. Without specific instruments to ensure action, calls for transformation of climate research, of climate action, and of society can remain ineffective, if not rhetorical or performative.

### Goals

Our general goal centers on a call for transformation in the way diverse knowledge systems serve to develop solutions-based research that can address the climate crisis, as they contribute to solutions to other, related crises; incremental change is insufficient to address longstanding inequalities and exclusions. This transformation rests on a rights-based justice approach, which entails mutual recognition and respect between different knowledge systems and which acknowledges that knowledge systems are intertwined with material and political systems—as shown in particular by the links that connect Indigenous knowledge with Indigenous land rights and self-determination. As stated in "Knowledge systems" section, this recognition entails the full acceptance of the complexity and breadth of knowledge systems. Indigenous knowledge systems and local knowledge systems are not restricted to observations, methodologies, and generalizations about the natural world; rather, they constitute entire ways of knowing—coherent, dynamic sets of practices, values, worldviews, and relationships, rooted in ties to land, language, and cosmologies.

### Decolonization

To move to full recognition of and respect between diverse knowledge systems requires addressing colonial relations in climate research. The transformational move to full inclusion of diverse knowledge systems and their holders involves confronting the power relations that have determined the historical undervaluing of certain groups and their knowledge systems. This process requires sustained efforts to decolonize research agendas and tools, toward the revitalization of Indigenous Peoples and of local communities, strengthening of their knowledge systems and ways of life, and protection of their lands and territories. Such decolonization must be based on respect for diverse worldviews (Mustonen 2013; Gilio-Whitaker 2019; Whyte 2017; Wilson et al. 2020; Callison 2021). As we discuss below, specific instruments at the international, national, and local scales can support processes of decolonization. In this way, they can move from the vertical and violent relations of colonialism and hegemonic Western worldviews to the horizontal and supportive relations of partnership between knowledge holders and worldviews, our second recommendation. As shown in "Transformative change" section, some of these instruments are not new, but they require broader and deeper application and fuller implementation.

### Partnership

The term partnership conveys a number of elements for the full inclusion of diverse knowledge systems. It indicates the importance of recognizing all knowledge holders as individuals, as communities, and as organizations. More than collaboration, partnership points to horizontal ties between equals. And more than co-production (see "Transformative change" section for a discussion of its meaning), partnership suggests ongoing ties and mutual responsibilities that extend over time rather than joint efforts on individual projects that are by nature limited in duration. Partnership also points to a fuller range of scales—from international and national to local—than co-production, which is often limited to smaller scales. Most importantly, partnerships respect the autonomy of diverse knowledge systems, rather than calling for their integration—usually a precursor of the subordination of other knowledge systems to scientific knowledge—or for the creation of a syncretic knowledge system that obliterates the distinctive nature of its constituents. Finally, partnership also implies sustained dialogue between two or more organizations or collectivities in ways that foster intercultural understanding. Such ongoing, sustained exchanges contribute to transformational rather than incremental change.

Partnership also implies a governance framework in which participants work together. As others have recognized (Carmona et al. 2022a; Pascual et al. 2022), governance to support just, equitable partnerships for climate research must function on multiple levels. At the international level, following the lead of biodiversity organizations—particularly the CBD and IPBES (Hill et al. 2020)—the UNFCCC must ensure that Indigenous Peoples engage in all of its subsidiary and constituted bodies. The IPCC should include more Indigenous authors and representatives of local communities in their assessments and convene Indigenous Peoples and other knowledge holders as official observers and reviewers (Ebhuoma and Leonard 2022; Lu 2022). At the national level, states should commit to and ensure the full participation of Indigenous Peoples in the design, monitoring, reporting, and verification of climate research and action, incorporating non-Western worldviews and forms of relating to nature. At the local level, all measures must be decided in accordance with the rights of the Indigenous Peoples involved; the implementation must respect land and resource tenure and local protocols, and must support as well the broader recognition of plural values and worldviews. For these governance measures to be successful, concrete steps must be taken to ensure that they lead directly to changed practices, leading to our third recommendation, implementation.

### Implementation

For governance mechanisms at local, national, and international scales to promote change—especially transformative change—they must link to and influence processes of implementation. These processes include the administration of rules and monitoring and evaluation of their application and processes for presenting grievances and adjudicating or arbitrating disputes. The diversity of knowledge systems involved in climate research partnerships places requirements of inclusivity and accountability on these processes of implementation. Since these knowledge systems include diverse types of relations between human communities and the non-human or natural world, these processes must ensure the respect of local worldviews, protocols, and customary practices. In turn, this mutual respect requires capacity building on all sides for the mutual understanding that is a necessary precondition for implementation, so that all parties are ready to undertake the collective work that constitutes ongoing partnerships. These processes of implementation take the concrete form of instruments, our fourth and final recommendation.

### Instruments

We note a number of existing instruments that can operate individually and jointly to advance the processes of decolonization. These processes, in turn, are necessary preconditions for genuine partnerships to achieve full inclusion of diverse knowledge systems into climate research. Indeed, the instruments serve not only to allow the sharing of knowledge but also the sharing of power. This power-sharing is a key element of the reconciliation that lies at the heart of decolonization.

An important element of the power-sharing lies in the continuous monitoring and evaluation of the instruments by the many parties involved, leading to learning and to evolution of the instruments. It cannot be assumed that the instruments, once established, will work so well that they can remain unchanged indefinitely. As shown by the history of instruments in "The position of diverse knowledge systems within international climate agreements and institutions" section, such monitoring and evaluation reveals gaps and deficits which can then lead to modifications in the instruments and the addition of other instruments.

We recognize that no set of instruments can guarantee justice and transformative change on their own. As discussed in "The position of diverse knowledge systems within international climate agreements and institutions" section, formal requirements for FPIC in the Philippines did not prevent mines and dams from being constructed in Indigenous lands, despite the opposition of the Indigenous Peoples. FPIC has had a checked history elsewhere as well; a review of 68 cases of consultations between mining firms and Indigenous and local communities across Latin America found some cases of success in supporting community efforts to protect their lands and other cases of failure (Walter and Urkidi 2017). Indeed, FPIC can have pernicious effects, by creating the appearance of consent when such consent is neither free nor informed, as was recently documented for the Ecuadorian Amazon (Etchart 2022).

However, though these instruments cannot be taken as a panacea, they are often powerful; the expansion of their use offers hope that transformative change is a genuine possibility. As the text box below shows, there has been some progress toward establishing these instruments through international agreements and toward implementing these instruments through governing authorities at national and local levels. We note that some of these instruments have existed and been included in principle for decades, but substantial gaps remain in terms of their prioritization and support that they have received—gaps that we signal in our discussions of decolonization and implementation above (Human Rights Watch 2019; Jong 2022).

We suggest that the expansion of such implementation, and indeed the expansion of these instruments, will promote the rapid movement toward transformative change that the current climate crisis requires.

### Policy instruments supporting partnership between diverse knowledge systems

- Full consultation. This instrument promotes the inclusion of Indigenous Peoples and of local communities in all stages of projects and programs, though it does not guarantee them an influence in shaping action. It supports transformative climate research by recognizing the rights to participation and self-governance. It is supported by the Indigenous and Tribal Peoples’ Convention of the International Labour Organisation (1989), as well as by operational policies of the Convention on Biological Diversity, the World Bank, a number of multilateral development banks, and a number of United Nations agencies. Examples of countries that have adopted consultation include India, Chile, and Brazil (Eimer and Bartels 2020; Tormos-Aponte 2021; Carmona 2022).
- Free, prior, and informed consent (FPIC). This instrument requires the full voluntary agreement of Indigenous Peoples or of local communities in advance of the implementation of projects or activities by outside parties that have the potential to affect them. It is therefore stronger than full consultation (since withholding consent can block projects), even though it is often only limited to the initial planning phases of projects. FPIC supports transformative climate research by recognizing the rights to self-determination and self-governance, as well as other related rights. It is supported by the United Nations Declaration of the Rights of Indigenous Peoples (2007). Examples include numerous countries in Asia, Africa, Latin America, and elsewhere (Williams and Hardison 2013; Godden and Tehan 2016; Papillon and Rodon 2017).
- Recognition of customary law. This instrument extends the first two instruments by granting full legal status to the traditional cultural rules and forms that regulate action; it includes all actions, rather than only the projects and programs as in the first two. It typically includes customary institutions that shape land- and water-based practices, particularly communal tenure and territorial rights. It supports transformative climate research by acknowledging and respecting the practices, values, and forms of relationality in Indigenous knowledge systems and local knowledge systems. It is supported by national legislation in some countries. Examples include national programs in Nepal, the Philippines, and Mexico (Rénique 2007; Sherpa et al. 2010; Poole 2011; CIPRED 2021).
- Intellectual property rights. This instrument requires researchers to recognize the ownership rights of knowledge holders to specific bodies of knowledge. It contributes to transformative climate research by guaranteeing the intellectual rights of all participants in partnerships. It is supported by the Nagoya Protocol of the Convention on Biodiversity (2014). In addition, in 2009, the Intergovernmental Committee on Intellectual Property and Genetic Resources, Traditional Knowledge and Folklore of the World Intellectual Property Organization began development of an international legal instrument to defend intellectual property rights for Indigenous knowledge systems and local knowledge systems; work on this instrument remains incomplete. Examples include Tanzania (PINGO’s Forum [Bibr CR104][Bibr CR105]; Climate Action Network Tanzania 2022; Lusiru and Malekela 2022; PINGO’s Forum 2022) and the US (Rimmer 2018).
- Indigenous data sovereignty. This instrument expands the scope of the previous instrument, intellectual property rights, by recognizing that intellectual property rights extend beyond the scope of specific bodies of knowledge to cover knowledge systems at large. The recognition of Indigenous data sovereignty supports transformative climate research by recognizing the rights of knowledge holders of diverse knowledge systems. It is supported by the proposed CARE and Fair Principles for Indigenous Data Futures (Carroll et al. 2020). Development of this instrument has advanced particularly in New Zealand (Cormack and Kukutai 2022; Reeves et al. 2022).
- Preservation and promotion of Indigenous languages. This instrument builds on the two previous instruments by supporting the languages that form the framework for the knowledge that those instruments address. The preservation and promotion of Indigenous languages supports transformative climate research by assuring the vitality and autonomy of languages that animate Indigenous knowledge systems (Basso 1996). It is often linked with education programs to assure the transmission of Indigenous languages. It is supported by the Los Pinos Declaration, the Outcome document of the high-level UNESCO event “Making a Decade of Action for Indigenous Languages” (2019). Examples include Peru and Fiji (Hornberger 2014; Eräsaari 2015; Linares 2017; Ocampo Yahuarcani et al. 2019; Harris et al. 2020; Gard 2021).

## Conclusion

As suggested above, the goals of transformation will not be achieved if they remain only as broad principles, since inevitably, disagreements will arise about how to put these principles into practice, and the forces that oppose these principles can use their breadth to delay or to block them. Bodies in public, private and non-profit sectors have time and again shown themselves to be adept in subverting instruments. We recognize that no call to action can offer a panacea, and that no system of prescriptions can guarantee full, just partnerships.

Nonetheless, we also recognize that the instruments have led to progress in a number of settings, as illustrated by the cases which we discussed at the close of "Knowledge systems" section and elsewhere in this article. Hence, we include a focus on implementation, and propose concrete instruments that can serve to accomplish this implementation. We recognize as well that further action is also needed to build genuine partnerships between the holders of diverse knowledge systems. The full use of the instruments discussed here can contribute significantly to this action. Without them, such action is likely to be very limited.

## References

[CR1] Adger WN, Barnett J, Brown K, Marshall N, O’Brien K (2013). Cultural dimensions of climate change impacts and adaptation. Nature Climate Change.

[CR2] Aikenhead G, Michell HJ (2011). Bridging cultures: scientific and indigenous and ways of knowing nature.

[CR3] Basso, K. H. 1996. Wisdom sits in places: landscape and language among the Western Apache. Albuquerque: University of New Mexico Press.

[CR4] Belfer E, Ford JD, Maillet M (2017). Representation of Indigenous peoples in climate change reporting. Climatic Change.

[CR5] Bezner Kerr, Rachel, Toshihiro Hasegawa, and Rodel Lasco. 2019. Chapter 5: food, fibre, and other ecosystem products. in *Climate change 2022: impacts, adaptation and vulnerability. Contribution of Working Group II to the Sixth Assessment Report of the Intergovernmental Panel on Climate Change*. Cambridge: Cambridge University Press.

[CR6] Blythe J, Silver J, Evans L, Armitage D, Bennett NJ, Moore M, Morrison TH, Brown K (2018). The dark side of transformation: latent risks in contemporary sustainability discourse. Antipode.

[CR7] Brugnach M, Craps M, Dewulf A (2017). Including indigenous peoples in climate change mitigation: addressing issues of scale, knowledge and power. Climatic Change.

[CR8] Callison C (2021). Refusing more empire: utility, colonialism, and Indigenous knowing. Climatic Change.

[CR9] Canada’s 2021 Nationally Determined Contribution Under the Paris Agreement. 2021. UNFCCC. https://unfccc.int/sites/default/files/NDC/2022-06/Canada%27s%20Enhanced%20NDC%20Submission1_FINAL%20EN.pdf

[CR10] Carmona, R. 2022. Pueblo mapuche, vulnerabilidad climática y política pública. Una aproximación desde la etnografía del Estado [Thesis for the degree of PhD in Anthropology, University of Bonn]. https://bonndoc.ulb.uni-bonn.de/xmlui/handle/20.500.11811/9569

[CR11] Carmona, R., J. Petrasek MacDonald, D. Sambo Dorough, T. Rai, G. Sanago, and S. Thorsell. 2022a. Recognising the contributions of Indigenous Peoples in global climate action? An analysis of the IPCC report on Impacts, Adaptation and Vulnerability. Copenhagen: International Work Group for Indigenous Affairs. https://iwgia.org/en/resources/publications/4621-iwgia-briefing-analysing-recognition-contrubutions-indigenous-peoples-ipcc-report.html

[CR12] Carmona, R., G. Reed, J. Ford, S. Thorsell, R. Yon, F. Carril, J. Cerda, and K. Pickering. 2022b. Recognition of indigenous peoples in nationally determined contributions. Copenhagen: International Work Group for Indigenous Affairs. https://iwgia.org/en/resources/publications.html

[CR13] Carroll SR, Garba I, Figueroa-Rodríguez OL, Holbrook J, Lovett R, Materechera S, Parsons M, Raseroka K, Rodriguez-Lonebear D, Rowe R, Sara R, Walker JD, Anderson J, Hudson M (2020). The CARE principles for indigenous data governance. Data Science Journal.

[CR14] Cartier, K. M. S. 2022. Maui Endures more drought and drier streams. *Eos*. https://eos.org/articles/maui-endures-more-drought-and-drier-streams

[CR15] Chakraborty R, Sherpa PY (2021). From climate adaptation to climate justice: critical reflections on the IPCC and Himalayan climate knowledges. Climatic Change.

[CR16] Chiblow S, Meighan PJ (2022). Language is land, land is language: the importance of Indigenous languages. Human Geography.

[CR17] Center for Indigenous Peoples' Research and Development (CIPRED). 2021. Indigenous Peoples’ Customary Governance for Sustainable Management of Natural Resources and Protection of Biodiversity. IUCN CEESP.

[CR18] Climate Action Network Tanzania. 2022. Climate services: come in out of the rain (summary for policymakers). https://cantz.or.tz/publications/15

[CR19] Cochran P, Huntington OH, Pungowiyi C, Tom S, Chapin FS, Huntington HP, Maynard NG, Trainor SF (2013). Indigenous frameworks for observing and responding to climate change in Alaska. Climatic Change.

[CR20] Cormack D, Kukutai T, Hepp A, Jarke J, Kramp L (2022). Indigenous peoples, data, and the coloniality of surveillance. New perspectives in critical data studies.

[CR21] Coscieme L, da Silva Hyldmo H, Fernández-Llamazares Á, Palomo I, Mwampamba TH, Selomane O, Sitas N, Jaureguiberry (2020). Multiple conceptualizations of nature are key to inclusivity and legitimacy in global environmental governance. Environmental Science & Policy.

[CR22] Davis H, Todd Z (2017). On the importance of a date, or, decolonizing the anthropocene. ACME.

[CR23] Daytec-Yañgot, C. L. 2012. FPIC: a shield or threat to indigenous peoples’ rights? https://www.un-redd.org/sites/default/files/2021-09/Indigenous%20Peoples%20Experiences%20on%20Free%2C%20Prior%20and%20Informed%20Consent%3A%20A%20Collection%20of%20Case%20Studies%28Philippines%29.pdf

[CR24] Díaz S, Demissew S, Carabias J, Joly C, Lonsdale M, Ash N, Larigauderie A, Adhikari JR (2015). The IPBES conceptual framework—connecting nature and people. Current Opinion in Environmental Sustainability.

[CR25] Douglas H (2000). Inductive risk and values in science. Philosophy of Science.

[CR26] Ebhuoma E, Leonard L (2022). Indigenous knowledge and climate governance: a Sub-Saharan African perspective.

[CR27] Eimer TR, Bartels T (2020). From consent to consultation: indigenous rights and the new environmental constitutionalism. Environmental Politics.

[CR28] Ellen R (2004). From ethno-science to science, or 'what the indigenous knowledge debate tells us about how scientists define their project'. Journal of Cognition and Culture.

[CR29] Eräsaari M (2015). The iTaukei chief: value and alterity in Verata. Journal De La Société Des Océanistes.

[CR30] Eriksen C, Hankins DL (2014). The retention, revival, and subjugation of indigenous fire knowledge through agency fire fighting in Eastern Australia and California. Society & Natural Resources.

[CR31] Etchart L (2022). Corporate social responsibility and the extractive industries in the Ecuadorian amazon: indigenous rights and the environment.

[CR32] Ferguson J, Weaselboy M (2020). Indigenous sustainable relations: considering land in language and language in land. Current Opinion in Environmental Sustainability.

[CR33] Fernández-Llamazares Á, Díaz-Reviriego I, Luz AC, Cabeza M, Pyhälä A, Reyes-García V (2015). Rapid ecosystem change challenges the adaptive capacity of local environmental knowledge. Global Environmental Change.

[CR34] Gard R, Luetz JM, Nunn PD (2021). Paddling on both sides of the canoe: toward a consilience of science and spirituality in climate change response. Beyond belief.

[CR35] Gilio-Whitaker D (2019). As long as grass grows: the indigenous fight for environmental justice, from colonization to Standing Rock.

[CR36] Godden L, Tehan M (2016). REDD+: climate justice and indigenous and local community rights in an era of climate disruption. Journal of Energy & Natural Resources Law.

[CR38] Graeber D, Wengrow D (2021). The dawn of everything: a new history of humanity.

[CR37] Gram-Hanssen I, Schafenacker N, Bentz J (2021). Decolonizing transformations through ‘right relations’. Sustainability Science.

[CR39] Granberg M, Glover L (2014). Adaptation and maladaptation in australian national climate change policy. Journal of Environmental Policy & Planning.

[CR40] Haag I, Kassam K-A, Senftl T, Zandler H, Samimi C (2021). Measurements meet human observations: integrating distinctive ways of knowing in the Pamir Mountains of Tajikistan to assess local climate change. Climatic Change.

[CR41] Haraway DJ (2016). Staying with the trouble: making kin in the Chthulucene.

[CR42] Harris, P., Brock, C., McInnes, E., Neill, B., Diamond, A., Carter, J., Camaitoga, U., Krishna, M., et al. (2020). Language and Literacy Learning in Multilingual Settings. In P. Harris, C. Brock, E. McInnes, B. Neill, A. Diamond, J. Carter, U. Camaitoga, M. Krishna, and E. Giannakis (eds), Children’s Multilingual Literacy (Vol. 31, pp. 41–75). Springer, Singapore

[CR43] Hill R, Adem Ç, Alangui WV, Molnár Z, Aumeeruddy-Thomas Y, Bridgewater P, Tengö M, Thaman R (2020). Working with Indigenous, local and scientific knowledge in assessments of nature and nature’s linkages with people. Current Opinion in Environmental Sustainability.

[CR44] Hornberger NH (2014). “Until I became a professional, I was not, consciously, indigenous”: one intercultural bilingual educator’s trajectory in indigenous language revitalization. Journal of Language, Identity & Education.

[CR45] Howe JP, Lightman B, Reidy MS (2014). Getting past the greenhouse: John Tyndall and the nineteenth-century history of climate change. The age of scientific naturalism.

[CR46] Huggel C, Bouwer LM, Juhola S, Mecler R, Muccione V, Orlove B, Wallimann-Helmer I (2022). The existential risk space of climate change. Climatic Change.

[CR47] Human Rights Watch. 2019. September 22. Indonesia: indigenous peoples losing their forests. https://www.hrw.org/news/2019/09/22/indonesia-indigenous-peoples-losing-their-forests

[CR146] International Labour Organisation (ILO). 1989. *Indigenous and Tribal Peoples Convention (Convention No. 169)*. Geneva: ILO.

[CR48] Ingold T (2011). Being alive: essays on movement, knowledge and description.

[CR49] Intergovernmental Panel on Climate Change (IPCC). 2014. Climate change 2014: mitigation of climate change: working group III contribution to the Fifth Assessment Report of the Intergovernmental Panel on Climate Change (O. Edenhofer, R. Pichs-Madruga, Y. Sokona, E. Farahani, S. Kadner, and K. Seyboth, Eds.). Cambridge: Cambridge University Press.

[CR50] Intergovernmental Panel on Climate Change (IPCC). 2018. Annex I: glossary (Matthews, JBR, Ed.). Cambridge: Cambridge University Press.

[CR51] Intergovernmental Panel on Climate Change (IPCC). 2022. Climate change 2022: impacts, adaptation and vulnerability. IPCC. https://www.ipcc.ch/report/ar6/wg2/

[CR52] Intergovernmental Science-Policy Platform on Biodiversity and Ecosystem Services (IPBES). 2017. Knowledge systems. IPBES Secretariat. http://ipbes.net/glossary/knowledge-systems

[CR53] Inuit Circumpolar Council (ICC). 2016. Application of indigenous knowledge in the arctic council. Anchorage, Alaska: Inuit Circumpolar Conference. https://iccalaska.org/wp-icc/wp-content/uploads/2016/03/Application-of-IK-in-the-Arctic-Council.pdf

[CR54] Ituarte-Lima, C., M. Schultz, T. Hahn, C. Mcdermott, R. Martinez-Peña, and S.E. Cornell. 2018. CBD voluntary guidelines for safeguards: implementation pathways. 10.13140/RG.2.2.24660.96643

[CR55] Jagannathan K, Arnott JC, Wyborn C, Klenk N, Mach KJ, Moss RH, Sjostrom KD (2020). Great expectations? Reconciling the aspiration, outcome, and possibility of co-production. Current Opinion in Environmental Sustainability.

[CR56] Jasanoff S (2004). States of knowledge: the co-production of science and social order.

[CR57] Jones R, Patwardhan A, Cohen S, Dessai S, Lammel A, Lempert R, Mirza M, von Storch H, Field CB, Barros VR, Dokken DJ, Mach KJ, Mastrandrea MD (2014). Foundations for decision making. Climate change 2014: impacts, adaptation, and vulnerability.

[CR58] Jong, H. N. 2022, August 1. No permit? No problem for palm oil company still clearing forest in Papua. Mongabay. https://news.mongabay.com/2022/08/no-permit-no-problem-for-palm-oil-company-still-clearing-forest-in-papua

[CR59] Kasdan M, Kuhl L, Kurukulasuriya P (2021). The evolution of transformational change in multilateral funds dedicated to financing adaptation to climate change. Climate and Development.

[CR60] Kassam K-A, Ruelle M, Haag I, Bulbulshoev U, Kaziev D, Louis L, Ullmann A, Edwards I, Khan AA, Trabucco A, Samimi C (2021). Engaging transformation: using seasonal rounds to anticipate climate change. Human Ecology.

[CR61] Kidd IJ, Medina J, Pohlhaus G (2017). Routledge handbook of epistemic injustice.

[CR62] Kimmerer, R. W. 2013. Braiding sweetgrass (First edition). Milkweed Editions.

[CR63] Klenk N, Fiume A, Meehan K, Gibbes C (2017). Local knowledge in climate adaptation research: moving knowledge frameworks from extraction to co-production. Wires Climate Change.

[CR64] Klinsky S, Meadowcroft J, Banister D, Holden E, Langhelle O, Linnerud K, Gilpin G (2019). Taming equity in multilateral climate politics: a shift from responsibilities to capacities. What next for sustainable development?.

[CR65] Krug CB, Sterling E, Cadman T, Geschke J, Drummond de Castro PF, Schliep R, Osemwegie I, Muller-Karger FE, Maraseni T (2020). Stakeholder participation in IPBES: connecting local environmental work with global decision making. Ecosystems and People.

[CR66] Kuyper JW, Linnér B, Schroeder H (2018). Non-state actors in hybrid global climate governance: justice, legitimacy, and effectiveness in a post-Paris era. Wires Climate Change.

[CR67] Lam DPM, Hinz E, Lang DJ, Tengö M, von Wehrden H, Martín-López B (2020). Indigenous and local knowledge in sustainability transformations research: a literature review. Ecology and Society.

[CR68] Latulippe N, Klenk N (2020). Making room and moving over: knowledge co-production, Indigenous knowledge sovereignty and the politics of global environmental change decision-making. Current Opinion in Environmental Sustainability.

[CR69] Levi-Strauss C (1966). The savage mind.

[CR70] Liboiron M, Tironi M, Calvillo N (2018). Toxic politics: acting in a permanently polluted world. Social Studies of Science.

[CR71] Linares RE (2017). Guided by care: teacher decision-making in a rural intercultural bilingual classroom in Peru. Intercultural Education.

[CR72] Longino HE (1990). Science as social knowledge: values and objectivity in scientific inquiry.

[CR73] Longino HE, Nelson LH, Nelson J (1996). Cognitive and non-cognitive values in science: rethinking the dichotomy. Feminism, science, and the philosophy of science.

[CR74] Lu, D. 2022. July 30. How scientists are working for greater inclusion of Indigenous knowledge. *The Guardian*. https://www.theguardian.com/australia-news/2022/jul/31/how-scientists-are-working-for-greater-inclusion-of-indigenous-knowledge

[CR75] Lusiru, S.N., and A.A. Malekela. 2022. Triangulating indigenous place names and meteorological data for a better understanding of climate change in same district, Tanzania. *International Journal of Environment and Climate Change*. 10.9734/ijecc/2022/v12i1131053

[CR76] Lyver PO, Timoti P, Gormley AM, Jones CJ, Richardson SJ, Tahi BL, Greenhalgh S (2017). Key Māori values strengthen the mapping of forest ecosystem services. Ecosystem Services.

[CR77] Maffi L, Callan H (2018). Biocultural diversity. The international encyclopedia of anthropology.

[CR78] McAllister TG, Naepi S, Wilson E, Hikuroa D, Walker LA (2022). Under-represented and overlooked: Māori and Pasifika scientists in Aotearoa New Zealand’s universities and crown-research institutes. Journal of the Royal Society of New Zealand.

[CR79] McKemey M, Rangers TB, Patterson M, Hunter J, Ridges M, Ens E, Miller C, Costello O, Reid N (2021). Indigenous cultural burning had less impact than wildfire on the threatened Backwater grevillea (*Grevillea scortechinii* subsp. Sarmentosa) while effectively decreasing fuel loads. International Journal of Wildland Fire.

[CR80] McMichael C, Katonivualiku M, Powell T (2019). Planned relocation and everyday agency in low-lying coastal villages in Fiji. The Geographical Journal.

[CR81] Mustonen T (2013). Rebirth of Indigenous Arctic Nations and polar resource management: critical perspectives from Siberia and Sámi areas of Finland. Biodiversity.

[CR82] Mustonen, T., Rivera Harper, M. Ferre, J.C. Postigo, A. Ayanlade, T. Benjaminsen, R. Morgan, A. Okem, et al. 2021. 2021 towards inclusion of indigenous knowledge and local knowledge in global reports on climate change. 10.13140/RG.2.2.14498.76485

[CR83] Nakashima, D., K. Galloway McLean, H. Thulstrup, A. Ramos Castillo, and J. Rubis. 2012. Weathering uncertainty: traditional knowledge for climate change assessment and adaptation. Darwin: UNESCO; UNU-IAS.

[CR84] Nakashima D, Nilsson A, Petitjean P, Zharov V, Glaser G, Richardson J, de Padirac B, Archibald G (2006). Linking biological and cultural diversity: Local and indigenous knowledge systems. Sixty years of science at UNESCO.

[CR85] Nakashima, D., J. Rubis, P. Bates, and B. Ávila. 2017. *Local knowledge, global goals.* Paris: UNESCO Document code:SC/UNESCO-LINKS/WS/2017, SC-2018/WS/https://unesdoc.unesco.org/ark:/48223/pf0000259599

[CR87] Nelson, M. 2020, December 22. Time to indigenize lands and water conservation. Sierra. https://www.sierraclub.org/sierra/2021-1-january-february/feature/time-indigenize-lands-and-water-conservation

[CR86] Nelson DJ, Madsen LD (2018). Representation of Native Americans in US science and engineering faculty. MRS Bulletin.

[CR88] Nelson MK, Shilling D (2018). Traditional ecological knowledge: learning from indigenous practices for environmental sustainability.

[CR89] New, M., Reckien, D., Viner, D., Adler, C., Cheong, S.-M., Conde, C., Constable, A., Coughlan de Perez, E., et al. (2022). Decision making options for managing risk. in climate change 2022: impacts, adaptation, and vulnerability: vol. Contribution of Working Group II to the sixth assessment report of the intergovernmental panel on climate change. Cambridge: Cambridge University Press. https://www.ipcc.ch/report/ar6/wg2/downloads

[CR90] Nightingale AJ, Eriksen S, Taylor M, Forsyth T, Pelling M, Newsham A, Boyd E, Brown K, Harvey B, Jones L, Bezner Kerr R, Mehta L, Naess LO, Ockwell D, Scoones I, Tanner T, Whitfield S (2020). Beyond technical fixes: climate solutions and the great derangement. Climate and Development.

[CR91] Nkuba MR, Chanda R, Mmopelwa G, Kato E, Mangheni MN, Lesolle D (2020). Influence of indigenous knowledge and scientific climate forecasts on arable farmers’ climate adaptation methods in the Rwenzori region Western Uganda. Environmental Management.

[CR92] O’Brien K (2012). Global environmental change II: from adaptation to deliberate transformation. Progress in Human Geography.

[CR93] O’Brien, K., C. Bethell, and T. Bjordam. 2021. You matter more than you think: Quantum social change for a thriving world. http://public.eblib.com/choice/PublicFullRecord.aspx?p=6788483

[CR94] O’Brien, K., and L. Sygna. 2013, June 19. Responding to climate change: the three spheres of transformation. Proceedings of Transformation in a Changing Climate.

[CR95] Ocampo Yahuarcani I, Jeri Lagos KD, Gutierrez Gomez E, Mendoza Valverde OM, Jeri Lagos LD, Saravia Llaja LA (2019). Educational tool for the teaching and self-learning of mathematics and language from mobile devices aimed at Quechua-speaking educational institutions of the initial level in Ayacucho, Peru. XIV Latin American Conference on Learning Technologies (LACLO).

[CR96] Okereke, C. 2018. Equity and justice in polycentric climate governance. In A. Jordan, D. Huitema, H. van Asselt, and J. Forster (Eds.), Governing climate change (1st ed., pp. 320–337). Cambridge: Cambridge University Press. 10.1017/9781108284646.019

[CR97] Oreskes N (2020). S*cience on a mission: how military funding shaped what we do and don’t know about the ocean*.

[CR98] Orlove B (2022). The concept of adaptation. Annual Review of Environment and Resources.

[CR99] Orlove B, Chiang JCH, Cane MA (2000). Forecasting Andean rainfall and crop yield from the influence of El Niño on Pleiades visibility. Nature.

[CR100] Orlove B, Shwom R, Markowitz E, Cheong S-M (2020). Climate decision-making. Annual Review of Environment and Resources.

[CR101] Papillon M, Rodon T (2017). Proponent-Indigenous agreements and the implementation of the right to free, prior, and informed consent in Canada. Environmental Impact Assessment Review.

[CR102] Pascual U, McElwee PD, Diamond SE, Ngo HT, Bai X, Cheung WWL, Lim M, Steiner N, Agard J, Donatti CI, Duarte CM, Leemans R, Managi S, Pires APF, Reyes-García V, Trisos C, Scholes RJ, Pörtner H-O (2022). Governing for transformative change across the biodiversity–climate–society nexus. BioScience.

[CR103] Petzold J, Andrews N, Ford JD, Hedemann C, Postigo JC (2020). Indigenous knowledge on climate change adaptation: a global evidence map of academic literature. Environmental Research Letters.

[CR104] PINGO’s Forum. 2021a. Climate change partnership with indigenous peoples in East Africa: indigenous knowledge and coping mechanisms. The Open Society Initiative for Eastern Africa. https://pingosforum.or.tz/wp-content/uploads/2021a/05/Indigenous-Knowledge-Coping-Mechanisms.pdf

[CR105] PINGO’s Forum. 2021b. Impacts of climate change mitigation strategies on indigenous peoples livelihoods: a case of Tanzania. The Open Society Initiative for Eastern Africa. https://pingosforum.or.tz/wp-content/uploads/2021b/05/Impacts-of-Climate-Change-Mitigation-Strategy-to-IPs.pdf

[CR106] PINGO’s Forum. 2022. Traditional knowledge system for agro-pastorialists resiliency. The Open Society Initiative for Eastern Africa. http://www.pingosforum.or.tz

[CR107] Poole D, Gotkowitz L (2011). Mestizaje, distinction, and cultural presence: the view from Oaxaca. Histories of race and racism: the andes and mesoamerica from colonial times to the present.

[CR108] Pörtner, Hans-Otto., Robert J. Scholes, John Agard, Emma Archer, Almut Arneth, Xuemei Bai, David Barnes, Michael Burrows, et al. 2021. Scientific outcome of the IPBES-IPCC co-sponsored workshop on biodiversity and climate change (Version 5). Zenodo. 10.5281/ZENODO.4659158.

[CR109] Raftopoulos M, Short D (2019). Implementing free prior and informed consent: The United Nations Declaration on the Rights of Indigenous Peoples (2007), the challenges of REDD+ and the case for the precautionary principle. The International Journal of Human Rights.

[CR110] Rarai A, Parsons M, Nursey-Bray M, Crease R (2022). Situating climate change adaptation within plural worlds: the role of Indigenous and local knowledge in Pentecost Island, Vanuatu. Environment and Planning e: Nature and Space.

[CR111] Reed G, Gobby J, Sinclair R, Ivey R, Matthews HD (2021). Indigenizing Climate Policy in Canada: A Critical Examination of the Pan-Canadian Framework and the ZéN RoadMap. Frontiers in Sustainable Cities.

[CR112] Reeves J, Treharne GJ, Theodore R, Edwards W, Ratima M, Poulton R (2022). Understanding the data-sharing debate in the context of Aotearoa/New Zealand: a narrative review on the perspectives of funders, publishers/journals, researchers, participants and Māori collectives. Kōtuitui: New Zealand Journal of Social Sciences Online.

[CR113] Rénique G (2007). Subaltern political formation and the struggle for autonomy in Oaxaca. Socialism and Democracy.

[CR114] Renn J (2020). The evolution of knowledge: rethinking science for the anthropocene. Princeton University Press.

[CR115] Reyes-García V, Fernández-Llamazares Á, Aumeeruddy-Thomas Y, Benyei P, Bussmann RW, Diamond SK, García-del-Amo D, Guadilla-Sáez S (2022). Recognizing Indigenous peoples’ and local communities’ rights and agency in the post-2020 Biodiversity Agenda. Ambio.

[CR116] Reyes-García, V., A. Tofighi-Niaki, B.J. Austin, P. Benyei, F. Danielsen, Á. Fernández-Llamazares, A. Sharma, R. Soleymani-Fard, et al. 2022. Data sovereignty in community-based environmental monitoring: toward equitable environmental data governance. *BioScience*, biac048. 10.1093/biosci/biac048 10.1093/biosci/biac048 PMC9343228 35923191

[CR117] Richardson, S.S. 2015. Sex itself: the search for male and female in the human genome (Paperback edition). The University of Chicago Press.

[CR118] Rimmer M, Rimmer M (2018). Northern exposure: Alaska, climate change, indigenous rights, and atmospheric trust litigation. Intellectual property and clean energy: the paris agreement and climate justice.

[CR119] Roesch-McNally G, Chang M, Dalton M, Lowe S, Luce C, May C, Morishima G, Mote P (2020). Beyond climate impacts: knowledge gaps and process-based reflection on preparing a regional chapter for the fourth national climate assessment. Weather, Climate, and Society.

[CR120] Salmon E (2000). Kincentric ecology: indigenous perceptions of the human-nature relationship. Ecological Applications.

[CR121] Schlingmann A, Graham S, Benyei P, Corbera E, Martinez Sanesteban I, Marelle A, Soleymani-Fard R, Reyes-García V (2021). Global patterns of adaptation to climate change by Indigenous Peoples and local communities. A systematic review. Current Opinion in Environmental Sustainability.

[CR122] Sherpa, P.D., K. Ghale, K. Lama, and P. Sherpa. 2010. Revitalizing customary governance and strengthening traditional knowledge on natural resource management in Nepal. In State of forests, policy environment & ways forward. Tebtebba Foundation.

[CR123] Shukla G, Kumar A, Pala NA, Chakravarty S (2016). Farmers perception and awareness of climate change: a case study from Kanchandzonga Biosphere Reserve, India. Environment, Development and Sustainability.

[CR124] Sillitoe, P. 2007. Local science vs. global science: approaches to indigenous knowledge in international development. New York: Berghahn Books.

[CR125] Talamayan F (2020). Mapping anti-dam movements: the politics of water reservoir construction and hydropower development projects in the Philippines. SSRN Electronic Journal.

[CR126] Tengö M, Hill R, Malmer P, Raymond CM, Spierenburg M, Danielsen F, Elmqvist T, Folke C (2017). Weaving knowledge systems in IPBES, CBD and beyond—Lessons learned for sustainability. Current Opinion in Environmental Sustainability.

[CR127] Thaman, R., P. Lyver, R. Mpande, E. Perez, J. Cariño, and K. Takeuchi. 2013. The contribution of indigenous and local knowledge systems to IPBES: building synergies with science. Report of the International Expert Workshop on the Contribution of Indigenous and Local Knowledge Systems to the Platform.

[CR128] Todd Z (2016). An indigenous feminist’s take on the ontological turn: ‘ontology’ is just another word for colonialism: an indigenous feminist’s take on the ontological turn. Journal of Historical Sociology.

[CR129] Tomlinson, M. 2020. Talanoa as dialogue and PTC’s role in creating conversation. *Pacific Journal of Theology*, 2.

[CR130] Tormos-Aponte F (2021). The influence of indigenous peoples in global climate governance. Current Opinion in Environmental Sustainability.

[CR131] Ulloa A (2017). Perspectives of environmental justices from indigenous peoples of latin america: a relational indigenous environmental justice. Environmental Justice.

[CR132] United Nations Educational, Scientific and Cultural Organization (UNESCO). 2020. Basic texts of the 2003 Convention for the Safeguarding of the Intangible Cultural Heritage. https://ich.unesco.org/en/basic-texts-00503

[CR133] United Nations Framework Convention on Climate Change (UNFCCC). 2022a. Santiago network for averting, minimizing and addressing loss and damage under the Warsaw International Mechanism for Loss and Damage associated with Climate Change Impacts. UNFCCC. https://unfccc.int/documents/624375

[CR134] United Nations Framework Convention on Climate Change (UNFCCC). 2022b. Report of the green climate fund to the conference of the parties and guidance to the green climate fund. UNFCCC.

[CR135] United Nations General Assembly (UNGA). 2007. 61/295. United Nations Declaration on the Rights of Indigenous Peoples (A/RES/61/295). UN. https://www.un.org/development/desa/indigenouspeoples/declaration-on-the-rights-of-indigenous-peoples.html

[CR136] van Bavel, B. 2021. Diversifying knowledge(s) to advance climate-health responses locally and globally [University of Leeds]. https://etheses.whiterose.ac.uk/29516/

[CR137] van Bavel B, MacDOnald JP, Dorough DS, de Pryck K, Hulme M (2022). Indigenous knowledge systems. A critical assessment of the intergovernmental panel on climate change.

[CR138] Vogel C, O’Brien K (2021). Getting to the heart of transformation. Sustainability Science.

[CR139] Walter M, Urkidi L (2017). Geoforum.

[CR140] Watson-Verran H, Turnbull D, Jasanoff S, Markle G, Pinch T, Petersen J (1995). Science and other indigenous knowledge systems. Handbook of science and technology studies.

[CR141] Whyte K (2017). Indigenous climate change studies: indigenizing futures, decolonizing the anthropocene. English Language Notes.

[CR142] Wildcat D, Montgomery M (2023). Traditional ecological knowledges: an antidote to destruction. Re-indigenizing ecological consciousness and the interconnectedness to indigenous identities.

[CR143] Williams T, Hardison P (2013). Culture, law, risk and governance: contexts of traditional knowledge in climate change adaptation. Climatic Change.

[CR144] Wilson KJ, Bell T, Arreak A, Koonoo B, Angnatsiak D, Ljubicic GJ (2020). Changing the role of non-Indigenous research partners in practice to support Inuit self-determination in research. Arctic Science.

[CR145] Yeh, E.T., 2016. ‘How can experience of local residents be “knowledge”?’ Challenges in interdisciplinary climate change research, *Area* 48: 34–40.

